# LONGITUDINAL STUDY OF ADULT SIBLING RELATIONSHIP AND PSYCHOLOGICAL WELL-BEING: THE ROLE OF CHILDHOOD MALTREATMENT

**DOI:** 10.1093/geroni/igad104.2170

**Published:** 2023-12-21

**Authors:** Jooyoung Kong, Kristin Homan, Jaime Goldberg

**Affiliations:** University of Wisconsin-Madison, Madison, Wisconsin, United States; Grove City College, Grove City, Pennsylvania, United States; University of Wisconsin-Madison , Madison, Wisconsin, United States

## Abstract

**Objectives:**

For many older adults, siblings serve as a source of companionship and support. This study examined a) the longitudinal association between sibling relationship quality and psychological well-being among older adults and b) whether the association would differ by childhood maltreatment.

**Methods:**

We used three-wave data from the Wisconsin Longitudinal Study. Latent growth curve modeling was conducted to examine the associations between perceived sibling closeness and the psychological well-being of 4,736 older adults.

**Results:**

Our findings showed that the trajectory of perceived sibling closeness differed between older adults who experienced childhood maltreatment and those who did not. Perceived sibling closeness was lower at baseline for those who experienced maltreatment and did not change over time, whereas perceived sibling closeness increased over time for those who did not experience maltreatment. Further, older adults who reported greater sibling closeness over time showed less of a decline in psychological well-being. The longitudinal associations between perceived sibling closeness and psychological well-being did not differ between the maltreated and non-maltreated groups.

**Discussion:**

Childhood maltreatment was found to have long-term negative effects on the emotional quality of sibling relationships in late adulthood. More research is needed to explore potential mechanisms that explain these lifelong associations.

